# The Syrian regime’s apparatus for systemic torture: A qualitative narrative study of testimonies from survivors

**DOI:** 10.1186/s12888-022-04425-w

**Published:** 2022-12-13

**Authors:** Niveen Rizkalla, Oussama Bakr, Sarah Alsamman, Salaam Sbini, Hana Masud, Steven P. Segal

**Affiliations:** 1 Mack Center on Mental Health & Social Conflict, School of Social Welfare, and Center for Effective Global Action, University of California Berkeley, 251 Giannini Hall, Berkeley, CA 94720-7400 USA; 2 School of Medicine, University of California Davis, 4610 X St, Sacramento, CA 95817 USA; 3 School of Medicine, University of California San Diego, 9500 Gilman Dr, La Jolla, CA 92093 USA; 4 Divinity School, Harvard University, 45 Francis Ave, Cambridge, MA 02138 USA; 5 Psychology Department, University of Massachusetts Boston, McCormack Hall, Boston, MA 02125 USA; 6 Professor of the Graduate Division and Chair Emeritus of the Mack Center on Mental Health & Social Conflict, School of Social Welfare, University of California, Berkeley, 120 Haviland Hall, Berkeley, CA 94720 USA

**Keywords:** Torture, Syria, Refugees, Captivity, Detention, Systemic Mechanism, Narratives, Testimonies

## Abstract

**Background:**

Despite broad interest of the Syrian refugee plight in the academic and media circles, there are still limited studies analyzing the lived experiences of torture survivors under the Syrian regime. This qualitative study interviewed torture survivors to examine the form and function of the Syrian regime’s security apparatus, and the personal aftermath of survivors.

**Methods:**

Thirteen in-depth interviews were conducted in Arabic with Syrian refugees who endured torture. Study participants were at least 19 years of age, resided as refugees in Jordan, and voluntarily agreed to participate in the study. Participation was anonymous and no incentives were provided. Only oral consent was required. Audio-recorded interviews were transcribed and translated to English, and then analyzed for repetitive themes utilizing the narrative approach.

**Results:**

Major themes were observed across three experience-phases: pre-captivity, during captivity, and post-captivity. The pre-captivity phase included two sub-themes: the Syrian regime’s initial detection and arrest system, and the intelligence system. The captivity phase was also divided into two sub-themes: environmental conditions in detention facilities, and torture methods including physical and psychological torture. Some of the environmental conditions in detention facilities included lack of sanitation, crowding, starvation, and withholding of medical care. Torture methods encompassed beatings, electric shocks, nail-pulling, hanging, drowning, suffocation, rape, and the witnessing of killing, sexual assault, or torture of others. The post-captivity phase included their release from captivity, escaping Syria, and post-displacement conditions and activism.

**Conclusions:**

The Syrian regime employs a vast security apparatus to track, detain, interrogate, torture, and subjugate its civilian population. A systematic mechanism commences even before captivity and continues for years after release, with negative implications on the well-being of survivors, their families, and the Syrian people as a collective community. The Syrian war saw a shift toward mass detention, torture as a form of social punishment, subjugation, and indeterminate imprisonment. Intervention agencies, host countries, and policymakers must be informed of survivors’ experiences to better address their needs. Moreover, the international community must advocate for a firm stance against torture, demand justice, and prosecute all parties engaged in perpetuating such extreme forms of suffering and trauma.

## Background

Despite international law in place to prevent cruel and inhumane treatment [1], torture remains a prevalent form of interrogation and punishment worldwide. In recent decades, human rights groups’ advocacy efforts have brought torture into the public eye. However, torture is not a modern practice. Its use in different forms dates back to ancient civilizations and has evolved over centuries. Torture in previous centuries took different forms and witnessed a shift in comparison to the current century [2].

Torture practices have increased in the twentieth century across Europe, Latin America, Asia, Africa, and the Middle East due to the rise of colonial, communist and fascist governments, and numerous international wars, which have been noted in the literature [3]. In response, the UN implemented the United Nations Convention against Torture and Other Cruel, Inhuman or Degrading Treatment or Punishment in 1987 [1]. Since then, 169 countries have ratified the convention to prevent cruel and degrading treatment and included torture as a criminal offense [4].

Torture remains prevalent in the twenty-first century across the global north and global south as witnessed in the aftermath of 9/11, the Arab Spring, and other international movements. However, unlike in earlier centuries when torture was publicly sanctioned by governments and legal systems, modern torture is clandestine, unregulated, and strongly denied by officials involved [3, 5, 6]. With human rights groups’ increasing documentation of torture practices, new torture methods were developed that leave no physical scars. Namely, electric shocks, sensory deprivation, sensory overload, temporal disorientation, and sleep deprivation [3, 5, 7]. Additionally, sexual humiliation and rape have been increasingly documented by human rights groups since the twentieth century [3].

## Syria’s torture apparatus

Despite ratifying various international human rights conventions during his rule, numerous human rights abuses were reported under former President Hafez al-Assad’s regime (1970–2000) [8]. The State of Emergency Law has been in place since 1963, which had expanded the regime’s executive authority to restrict civilians’ freedom to meet and travel, censor letters and communication, and arbitrarily arrest and detain thousands without trial for indefinite periods [8, 9]. Torture methods inflicted upon these detainees included, but were not limited to: suspension by a *dulab* (tire) and beating with sticks, *shabeh* (hanging) from the ceiling, electric cables, or whips, nail pulling, burning with cigarettes, beating the soles of the feet while being strapped to a table (*falaqa*), strapping to a piece of foldable wood and folding the victim within the wooden panel while applying electricity shocks (*bisat al-rih* or “the flying carpet”), applying electric shocks or heated metal to genital and anal regions, torturing other prisoners in front of the detainee/s, and threatening to torture and/or sexually assault detainees’ family members [9].

Holding on centralizing power in the presidency with entrenched brutal force, Hafez al-Assad’s regime silenced opposition through killing 1,000 detainees in Tadmor Palmyra Prison after the president’s failed assassination attempt, and the 1982 Hama massacre, wherein regime forces encircled and killed over 10,000 civilians in the city of Hama, a stronghold of the Muslim Brotherhood, which opposed the regime [10]. Other fatality estimates reported a range between 2,000 to 40,000 killed civilians [11]. This massacre was followed by mass arrests of over 100,000 people, effectively crushing any remaining political dissent [10].

After taking over his father’s regime in 2000, Bashar al-Assad implemented similar tactics to suppress dissent against his regime and kept the detention system in place. Individuals suspected of harboring anti-regime views faced enforced disappearances, arbitrary arrests, detention without trial, and torture in detention facilities [12]. In addition to previous torture practices such as the *falaqa*, the *dulab*, and *bisat al-rih, shabeh,* rape and sexual assaults, threats to rape relatives, witnessing and hearing torture of others, detainees were also subjected to “welcome party beatings” upon arrival to facilities, diverse positional abuse methods such as *al-kursi al-almani* or "the German Chair” (strapping the arms and legs to a metal chair whose back is adjusted backwards abruptly, often resulting in permanent injury or spinal damage), and *al salb* or “crucifixion” (hands and feet tied to a cross while beating is applied to the reproductive organs), as well as burning, hot/cold water pouring, drowning, suffocation, electric shocks while standing in water, solitary confinement, overcrowded prisons, and mock executions [12–14].

Decades of oppression ignited by the Arab Spring, anti-government protests began in March 2011 in response to the Syrian regime infuriating action of seizing and torturing 15 schoolboys from Daraa for spray painting “The people demand the fall of the regime” on their school walls [10]. President Bashar al-Assad’s response to political dissent during the Arab Spring, however, was not unlike that of his father and former President, Hafez al-Assad [8]. Subsequent demonstrations turned violent as the Syrian regime’s security forces conducted mass arbitrary arrests and fired live ammunition, killing protestors. Beginning in April 2011, security forces were ordered to shoot to kill in cities where anti-government demonstrations were taking place. As internal resistance to killing civilians grew within the Syrian army, army defectors and civilians formed the Free Syrian Army, transforming the opposition from peaceful demonstrations to an armed struggle against Bashar al-Assad regime [15].

Currently, the detention and torture system is an expansive version of the one built by Hafez al-Assad as it has been maintained, primed, and matured by Bashar al-Assad. In the last decade since the war’s eruption, the regime has utilized an intelligence system comprising of four agencies—military, political, air force, and state security, and their hundreds of local branches—to implement mass detentions, accelerate the killing of citizens under torture, and increase torture brutality. Many detainees went through an odyssey of torture facilities, each with its own notorious reputation [16]. For example, Branch 215 is called “Branch of Death”, whereas the Sednaya prison is known as an “extermination” prison [17]. When detainees were transferred to Sednaya Military Prison, they faced a one to three-minute trial in a military field court and were sentenced based on their confession under torture [18]. Those sentenced to death were executed in mass hangings, an average of 20 to 50 hanged prisoners each week, who were disposed of in a crematorium where detainees’ remains were buried in mass graves [18, 19]. It is estimated that these torture practices and extrajudicial executions have killed 5000 to 13,000 people in Saydnaya prison alone between 2011 and 2015 [12, 19]. Over an approximate of 100,000 remain in captivity in Syrian detention facilities [20], whereas at least 75,000 were reported missing between 2011 and 2016 [21]. By all accounts, the detention system is overwhelmed, with uncounted dungeons in non-conventional locations to contain the formidable number of detainees [16].

Despite the growing body of literature on Syrian refugees’ traumatic experiences, first-hand empirical research on the lived experiences of torture survivors specifically is still scarce. The complexity involved in accessing refugees and gathering information about their torture history, especially given Syrian survivors’ particular vulnerability and the stealthy and secretive torture apparatus of the regime, pose challenges to studying the human rights violations and abuses experienced. However, given the significant impact of torture on survivors’ well-being, it is essential that their experiences are better understood by policymakers and humanitarian agencies serving these populations and regions. Thus, to address this knowledge gap, this research aims to explore the subjective narratives of Syrian torture survivors, and their experiences before, during and in the aftermath of captivity in Syrian detention facilities.

## Methods

This study is part of a larger research project that explored Syrian refugees’ physical and mental health needs, and the humanitarian aid workers who assisted them. It aimed to explore Syrian torture survivors’ subjective narratives, with the purpose of shedding light on their personal experiences from the moment of detection (including arrest, detention, torture, release, and escape from Syria) to the phase of post-displacement. In conducting this qualitative research using the narrative paradigm, the team of researchers in this study hoped to provide a space for survivors to voice their unheard and unacknowledged experiences [22, 23]. The narrative research aims to unravel the life stories of people as they were told by them in their own words and worlds, which capture the complexities and nuances of their significant experiences [22]. Through storytelling, people order their life experiences and provide personal meaning to them [24]. Thus, this research method is a person-oriented approach, in which the narrators construct and compose their stories in the social, cultural, and lingual contexts as they perceive them [22]. This study utilized narrative research as its methodological framework in both data collection and interpretive or analytical processes to understand survivors’ sensemaking and life choices. It considered temporality, sociality, and place. Meaning that researchers focused on chronologically organizing survivors’ narratives into past, present, and future (temporality), as well as personal and social conditions under which experiences and events occurred (sociality), and the physical space where events took place [22, 24]. Although the sociality aspect of the narrative research considers both participants and researchers as working collaboratively in constructing meaning of the phenomena studied, researchers on this paper chose to mainly focus on participants’ lived experiences as the leaders of their own stories. The interview process did not include any assessment or evaluation of survivors’ physical or mental health state and thus researchers analyzed the narratives according to the way participants perceived and experienced them and without questioning the “truth” or extent of “objectivity” of their stories. It was hoped that utilizing the narrative approach in the interviewing process would be perceived as empowering and healing, in allowing participants to take agency and ownership on the events that happened to them and in the way they chose to present them to the public eyes.

## Recruitment

The first author partnered with humanitarian organizations serving Syrian refugees in Jordan to recruit study participants. To enable a safe space, the organizations offered their local facilities to conduct in-person interviews, and their staff invited known refugees to participate in this study. Initially, Syrian refugee women were among the first who agreed to take part in the interviewing process. However, during their interviews, women recounted horrifying narratives of torture experienced by their family members and acquaintances, which prompted further investigation. The study was thus modified to include both male and female Syrian refugees who had survived torture. These additional participants were recruited through study participants’ and organization volunteers’ personal connections, via a snowball sampling recruitment process. Subsequent interviews took place during the afternoon in either public spaces or at the participants’ homes. The place and time of interviews were determined according to participants’ preference and due to feelings of suspicion towards a stranger researcher, and their need to protect themselves and their families as they still feared from being targeted.

Participants included in the study met the following criteria: being a Syrian refugee who survived torture in Syria, at least 19 years of age, residing in Jordan during the data collection, and willing to participate in the study. Participation was voluntary, anonymous and no incentives were provided. For participants being interviewed in public spaces, the researcher offered food and beverage following regular hospitality and social norms of the region.

## Data collection

Between May to June 2014, the first author conducted in-depth interviews with Syrian refugees in Arabic. Most interviews lasted between 1.5 and 4.5 h, with one 6.5-h long interview. Demographic information—age, marital status, city of residence in Syria, and number of children—was obtained at the beginning of each interview. The remainder of the interview followed a semi-structured format with open-ended questions that eventually developed into an in-depth ethnographic interview, in which participants shared detailed personal narratives on their life before, during, and after captivity in response to the researcher’s request: “Tell me about your torture experience with any information you feel comfortable in sharing.” Despite the topic’s sensitivity, participants established a trusting relationship with the researcher and shared their extremely traumatic experiences [25]. Given participants’ emotional states after recounting traumatic experiences, the researcher closed interviews with a positive tone, inquiring about coping strategies and support systems. In cases when no support systems were disclosed, the researcher referred participants to humanitarian agencies that are specialized in assisting survivors with their healing process and coping. Additionally, the researcher stayed in touch with participants during her stay in Jordan and checked how they felt after the interviews. She continues being in touch with participants years after her departure to the USA.

During the interview process, the researcher responded to each participant’s narrative and emotions, and frequently clarified her understanding of participants’ narrations due to linguistic differences in Arabic dialects, or whenever she encountered an unfamiliar topic [24, 26, 27]. Although both researcher and participants spoke Arabic and shared similar social and cultural norms, the researcher’s Palestinian Arabic dialect was slightly different than the participants’ Syrian dialect, in addition to a few differences in specific regional norms and customs, which required clarifications when needed. These clarifications were perceived with humor and appreciation and created an atmosphere of closeness and solidarity during the interviews.

## Ethics

The Committee for the Protection of Human Subjects at University of California Berkeley approved this study with all its procedures, including a waiver for a written informed consent (CPHS, February 2014). Each participant received a consent form and sheet detailing the study’s aims and protocol prior to their participation. Only oral consent was required from participants. Permission to audio record interviews was also obtained after informing participants of how their quotes would be utilized anonymously during dissemination of this study. Only one participant refused to be audio-recorded and instead notes were taken during the interview. Participants were reassured that they could refuse to answer any question, withdraw from the study, and/or stop the audio-recording at any time. Participants were also notified that, if they were harming themselves or others, or at risk of doing so, the researcher would have to report the case to authorized organizations. To protect confidentiality, the researcher requested participants to not mention their own names or any other pieces of identifying information during interviews. The researcher addressed participants via pseudonyms of their choice. Prior to the transcription and translation of audio-recordings, the audio files were computer password-protected and labelled numerically to distinguish them from one another. All mentioned identifying demographics and pseudonyms used in the interviewing and analysis processes were removed from this manuscript, in the purpose of protecting participants’ privacy and preventing any risk of mistaken disclosure of their personal information.

## Data analysis

Interview audio-recordings were transcribed, translated, and analyzed by a team of five researchers who are Arabic and English native speakers, and who are of Syrian and Palestinian ethnic backgrounds. The first author edited interview transcripts and included non-verbal cues such as participants’ tone, pauses, crying, and narration tempo [24]. A sixth researcher participated in the data analysis and manuscript preparation but wished to remain anonymous as a precautionary measure. The seventh senior researcher assisted in study design, funding, and manuscript preparation. Academic backgrounds of these researchers included mental health, medicine, and theology/religious studies. Each researcher independently read interview transcripts and identified major repetitive themes. In subsequent group discussions, researchers deliberated on themes that they identified until reaching a consensus on major themes derived from the transcripts [28]. These themes were documented in an index book with assigned colors. Researchers then independently coded interview transcripts line by line according to the index book. Afterwards, they compared differences in their coded transcripts and resolved disagreements by discussing and accepting the majority opinion on final coded analysis [29–31].

Researchers added themes and sub-themes to better represent and capture the nuance of the participants’ narratives during the analysis process [24]. The interview transcription, translation, editing, and analysis process lasted 3.5 years. Researchers were exposed to extremely traumatizing descriptions of torture, and often needed to distance from the interviews for some time before returning to their research work [32]. Further delays were caused by long discussions among team members regarding the risk of publishing this research to themselves, their families and relatives residing in Syria [33]. These unusual challenges faced by the team are brought here for learning purposes to be taken into consideration by any academic researchers who intend to undertake similar complex projects.

## Results

### Sample and participants

Thirteen Syrians (ten men and three women) participated in this qualitative study. All endured and survived torture by the Syrian regime during the war that erupted in 2011, and some had additionally experienced torture in the years prior to 2011. At the time of the interview, participants resided in the urban areas of Jordan – namely, Amman, Irbid, and Ar-Ramtha. They have resided in Jordan from 4 months to 2 years (M = 13 months, SD = 5.67 months). Prior to their escape from Syria, participants have resided in Daraa, Ar Ruhaybah, Homs, Damascus, Al Moadamyeh, and Idlib. Age ranged from 22 to 50 years old (M = 36.08, SD = 10.20). The majority were married (53.85%), whereas 30.77% were single, and 15.38% were divorced. Married and divorced participants had one to five children (M = 3, SD = 1.31). Educational level was as follows: 7.69% had some school, 23.08% had finalized high-school, 38.46% had some college, and 30.77% finished college or higher.

Analysis of interviews identified many recurrent themes, which were divided into three experience-phases: pre-captivity, during captivity, and post-captivity (Table 1). In pre-captivity, two sub-themes were distinguished: an initial detection and arrest system; and the intelligence system. In this phase participants described how the Syrian regime thoroughly investigated their personal and professional lives or their involvement in the revolution to later justify an arrest, as well as narrated on the different facilities utilized within the intelligence system. During captivity, two sub-themes were also identified: environmental conditions and torture methods. Horrendous environmental conditions included overcrowding, sleep deprivation, starvation or poor food quality, dreadful sanitation and hygiene conditions, extreme temperatures, lack of light and ventilation, exposure to sounds of others being tortured, and the withholding of basic medical care. Torture methods encompassed various forms utilized to inflict physical and psychological suffering. Among a few were aggressive interrogations, humiliation, beatings (to the extent of multiple injuries, extensive bleeding, broken bones, miscarriages, etc.), electric shocks, solitary confinement, body mutilation, nail pulling, hanging, drowning, suffocation, rape, and witnessing others being tortured, raped, sexually mutilated, and killed. The third phase, post-captivity and the aftermath of torture, included descriptions of their release from captivity, escape from Syria, post-displacement life in Jordan, and post-captivity political activism.

**Table 1:** Major themes and sub-themes of survivors’ torture testimonies

**Major themes**			
	**A. Pre-captivity**	**B. Captivity**	**C. Post-captivity**
**Sub-themes**	**1. An initial detection and arrest system**	**1. Conditions in captivity**	Release from captivity
	A regime that has “ears and arms everywhere”	**2. Torture methods**	Unreleased captives after enforced disappearance
	Location of arrests	Physical torture	Feelings upon release
	Transfer to detention facilities and the ‘welcoming party’ reception	Psychological torture: Threats and involvement of family and friends	Escaping Syria
	False accusations	Depersonalization, derealization, and solitary confinement	Life in Jordan
	**2. The intelligence system**	Positional torture—Stress position abuse	Political activism
	Transfers between facilities	Al-Shabeh—Hanging	
	Sednaya: The ‘silent prison’, the ‘last stop’ or ‘extermination’ prison	Rape by interrogators	
	Adra central prison for women	Prisoners forced to rape other prisoners	
	Political intelligence and military intelligence branches	Killing of prisoners	
		Health related torture	

A word of caution to readers: The following results section contains detailed narratives and descriptions of events that participants have experienced. These descriptions are presented as testimonies and direct quotes in the form participants contextualized and wished for the world to hear and acknowledge them. The authors of this manuscript did not make any changes to these quotes to remain respectful of participants’ wishes, which also align with the narrative research approach in focusing on presenting the voices and stories of participants as they were narrated. Thus, readers need to know that these testimonies contain aggressive, brutal, and traumatizing descriptions that might be difficult to read and cause discomfort and distress.

#### Pre-captivity

**An initial detection and arrest system** Survivors had one or more encounters with the Syrian regime’s security apparatus prior to being captured and tortured. Extrajudicial arrests and brutal transfer to detention facilities occurred without formal, legitimate legal charges being applied. Following their arrests, survivors described similar patterns of interrogations and fabrication of claims.

*A regime that has “ears and arms everywhere”* Survivors believed and repeatedly referenced this phrase that represents and illustrates the country-wide fear and mistrust of the regime. It alludes to the idea that the regime uses its omnipresent network of informants, who can potentially be any citizen in their surroundings whom they force to collaborate with their detection system, thus even the walls have ears that can hear. One participant explained the oppressive effects of this threat: “*He [al-Assad] made people so ignorant, and he deprived people so much, and he prevented them, and [he] made them scared of even the walls. There is a saying ‘the walls have ears’ to such an extent that a person doesn’t dare speak [negatively about the government] to his friend because they are both scared that the walls would be listening to them*” (age range 20–30). A similar saying was used by another participant: “*Because we shut our mouths, and the walls have ears… That’s how we’re living [in Syria]. The walls have ears*” (age range 40–50). One survivor identified how informants actions led to mass arrests during the Syrian War: “*Raiding operations… Came in to catch young men… They have many informants, everything reaches them… Their eyes are extensively spread.”* This same survivor also explained his point of view on how the regime perceives its strategy of information-gathering as crucial for its survival: *“The security force knows all the details of each person’s personality, they count his breaths, I mean that they know the situation of every citizen, [including] where he works, where he is staying up late, how much money he earns… They know everything, their theory is that with such a method they will continue ruling the country till the Day of Judgment*” (age range 40–50).

*Location of arrests* Some survivors were arrested at their homes, whereas others were in public places at the time of their arrest. However, most survivors experienced ‘enforced disappearance,’ meaning they disappeared or were abducted without leaving any trace, suggesting they were taken by authorities. One participant described how the regime raided his home during his first arrest: “*I was sitting in my house, sleeping. With my family, my wife and I have two daughters. I didn’t know anything. Cars came, they surrounded the house… Right away they got me to the ground and started hitting me… They handcuffed me and so…*” He also described the prevalence of Syrians being suddenly kidnapped and the difficulty for families to obtain information from authorities on the fate and whereabouts of their missing family members “*You disappear and no one knows where you are or what happened to you*” (age range 30–40). Others were victims of mass arrests at places of worship. Houses of worship in the Middle East are religiously and culturally considered protected and may provide shelter at times of war due to their sacred value. Thus, invasions with acts of violence are perceived as deviation from religion teachings as well as violation of international agreements: “*They started to catch people and use an electric taser baton on their heads… They put them into cars and tied the people up. They started a mass arrest of the people who were in the mosque*” (age range 40–50). Another participant was anxious about compromising the wellbeing of his daughters who were left alone in the car at the time of his second arrest. Such arrest shattered the intimate and private space of the family and wounded the masculine pride of a father in front of his daughters when he was prevented from protecting them: “*My daughters were in the car and there was no one in the street… I told the soldiers, ‘I have the girls. Let me just tell them. Let me get someone to get them out of the car and take them home. It’s about to get dark.’ It’s a checkpoint, once it gets dark the street is very empty, nobody would pass by. And the soldier started yelling, ‘Sit down idiot. Shut up idiot. Damn you and your daughters… Why are you going to ever see your daughters again? You are going to be executed’… They were cursing and yelling… ‘You are going to your death.’ And my mind was just with my daughters. They were left in the street. They were still in the car*” (age range 30–40). One survivor was only a student when his name appeared on a security blacklist, leading to his immediate detainment when he was stopped at a checkpoint: “*They took me from the borders to the intelligence branch of Daraa. I was coming from Jordan to Syria in a taxi*” (age range 20–30).

*Transfer to detention facilities and the ‘welcoming party’ reception* Captors first bound and blindfolded those they arrested, followed by transportation to the detention facilities, which involved brutal treatment. Upon arrival, guards carried out an indiscriminate beating referred to as a ‘reception’ or ‘welcoming,’ as part of their standard procedure. One participant recounted: “*They started to grab us from our hair and legs, and they threw us like this [as if we were nothing]. Stairs… I do not know… There were a lot of people, and maybe it was because people were underneath me, or because it was cold weather, and we were freezing. I did not know what we were colliding with. And we were blindfolded, I could not see a thing, and no one could see. They did not even know our names until the officer who had the list of names came. They threw us and they started beating us… With batons/sticks, with belts, with chains… I do not know. A lot of beating, beating, beating, beating, beating. The smart one was the one who could [hide] himself*” (age range 40–50). Another participant described his arrival to a new facility: “*This type of thing happened only the first couple of hours, as a reception. We always had something called a reception… A reception party happens with hitting and beating… They have it as a party… This was the norm*” (age range 20–30).

*False accusations* Several survivors were not given an official reason for their arrests until after detention. Fabricated claims were sometimes presented only after some period of detention or torture to exert pressure on detainees into revealing what they have committed and whether the accusations were true or false. A participant was coerced to accept such claims days after his arrest: “*On day eleven [of daily torture]… I had to sign on claims [he did not commit]*” (age range 20–30). Another participant described the crimes she was accused of: “*They accused us of knowing armed gangs, that we provide medicine and weapons to [these armed gangs], and that wasn’t true. What would happen is, if I was walking in the street and found someone who was wounded or injured, I would take them in my car to the Maidani Hospital near my house, and that was that. And, if there was someone who needed food, I would help them with that. But weapons-this and weapons-that; it’s all not true*” (age range 40–50). Such false accusations align with Physicians for Human Rights’ reports on health workers who courageously adhered to their professional ethics, and provided care to the sick and wounded, which resulted in active persecution, detainment, and torture [34].

**The intelligence system** The intelligence system comprises four agencies: military, political, air force, and state security, which are subdivided into local branches and departments with facilities throughout Syria. Some branches are named after their location or specialization while others have earned their names due to historical roles. Survivors shared common notions regarding the infrastructure of the Syrian detention system. Several survivors were transferred between multiple branches as part of an intentional tool utilized to cause confusion, insecurity, and further psychological torment. The reputations and specializations of the infamous branches were also repeatedly mentioned.

*Transfers between facilities* Survivors who were accused of multiple charges were wanted for investigation at multiple branches, so they served a series of detention stays. One of the participants was detained at many facilities without being allowed to know the locations or durations of his detentions: “*After I finished with them [initial branch], they transferred me to another branch, which was the ‘Mukhabarat Askariya’ [Military Intelligence]. After that, they transferred me to Political Intelligence. After Political Intelligence, they transferred me to Criminal Intelligence. After Criminal Intelligence, they transferred me to Homeland Security… So, I would stay with each one for two months… And I would eat my share [of torture]… I didn’t know all of this back then. For example, when I was in the political branch, I didn’t know I was in the political branch—I didn’t know. And [when I was] in the criminal branch, I didn’t know I was in the criminal branch. And [when I was] in the homeland security branch, I didn’t know I was [there].*” He also compared the transfer system to a train with a sequence of stops: “*Each branch has its own lists [of wanted people], and [it was known] which branch is looking for whom. These lists are distributed… It’s a chain of branches… A sequence of stops. It’s a train and it stops at every stop… I’m riding the train and they would drop me off at every stop. They showed me its buffet and they showed me its toilet [filth], and then they returned me to the train*” (age range 40–50). During a transfer, another participant was led to believe he was about to be released, only to discover he was actually being transferred for further detainment: “*Al-Fayhaa branch, the political security branch in Fayhaa… The thing that affected me and killed me the most was that… He [the guard] told us, ‘Come on, get ready. You are going home to your families.’ [We told him], ‘Seriously? God bless you…’ I didn’t leave anything [thankful] that I didn’t say, and I would kiss him… [because] I had lost hope. I felt like I had died. So, I [now] became optimistic… On the way to Damascus, they transferred me from Daraa to Damascus. We thought we were going to the court. At the court, we [thought we] would sign a pledge and then get out. [Instead,] what were the soldiers saying among themselves? ‘You dogs! By God we are going to slaughter you!’ And we said, ‘But sir, we are going home.’ They said, ‘Where are you going? To which home?’ We said, ‘To the court.’ And he [the soldier] replied, ‘Oh really? I’m taking you to the court? Oh yes, to the court of death’ [mocking]*” (age range 20–30).

*Sednaya: The ‘silent prison’, the ‘last stop’ or ‘extermination’ prison* Two participants were detained in the Sednaya prison, notorious for being the “last stop”, where captives spend their last days in silence (talking is forbidden) before execution. The first participant described its reputation: “*Damascus [prison] is for execution. This is known… We would say if they only left us a year in there [jail] then bring us to Damascus. We know that in Damascus—that’s it—you are done… They would have transferred me to Damascus. From Daraa to Damascus. This was for soldier abduction [the accusation]. This charge is punished by execution. They sent us to Damascus. We thought we were being released [home] and we were so happy… We got out [of the cars] in Damascus. In a branch, it was called the Fayhaa Branch—it is well known in Damascus. They bring everyone [arrested] in Syria here [this prison]… We had old people with us as well… And the rest of us were young men. There were eight of us. As soon as we got there, they made us take off all our clothes. There you are completely naked*” (age range 30–40). The second participant’s transfer to Damascus also illustrated the prison’s reputation: “*We arrived to Damascus, to the city… On the way to Damascus there was a battle on the highway, on the international Daraa highway, from Daraa to Damascus [highway]. I said ‘Oh god, I hope a couple of guys would show up and spray [with shooting] the soldiers and us. And we die, it was enough.’ When we were transferred to Damascus, imagine our spirits [morale]. We were supposed to go home, but they [instead] just transferred us to a different branch, the most difficult prison in Syria*” (age range 20–30).

*Adra central prison for women* Adra central prison, a facility for female captives, was relatively less known by survivors before being imprisoned at the site. Two participants expected a milder treatment at this facility. The first participant described her initial thoughts about her upcoming transfer to Adra central prison: “*They wanted to transfer me from the branch to the prison, of course there [Adra] is much easier [than the branch], I said [to myself] ‘what a relief [farajallah], how adorable that is’, I was so happy, I am about to leave torture [which she soon discovered was not the case]*” (age range 40–50). The second also survived torture in this facility but witnessed additional unique conditions: “*In the Adra prison, there is no torture [sarcasm], but if someone is missing something [criminal behavior] one would learn it in there. For example, if they put someone in jail because he was caught using drugs [false accusation]… And inside the prison there are drugs… Like they would learn what is missing [criminal behavior]*” (age range 40–50). Both survivors’ expectations were dissolved after their stay at Adra central prison. Not only this prison included torture and maltreatment of captives, but it also taught captives how to survive utilizing criminal behavior, drug usage, and other felonies.

*Political intelligence and military intelligence branches* Various branches of the political intelligence and military intelligence apparatuses were named during interviews as survivors made references to their infamous torture methods. Reflecting his fear of these systems, one survivor was grateful to have avoided a full detention at one facility: “*Under the military—I was detained for only a few hours. Of course, here I had an intermediator [wasta] right away. If I didn’t have an intermediator [wasta] I would have been tortured and this one—the political security branch is known to be the best branch [as in the least harsh]. But the military [branch], if I had gone to it, I would have died from the beating. I was released—I finished from the military security. They interrogated me—a normal interrogation—and I left*” (age range 30–40). Another participant described the relationship between these intelligence networks and allied militias: “*The department of military security in Daraa, the city of Daraa, which follows [belongs to] the military security branch in Suwayda. The leadership branch in As Suwayda. The person in charge of it is the colonel Wafiq al-Naser. He is among the people in charge of all the crimes in Daraa. He is the one in charge of Daraa’s case. They welcomed them, the committees’ militias [shabeha], who are the national army, and who are civilians volunteering in the army, Halish [The Lebanese Shiite Hezbollah] and Maish [Non-Lebanese Shiite organizations supported by Iran] and stories like this [fighting in Syria and supporting the regime]. Same thing [happened], they [regime guards] raped them [group of young women] five times*” (age range 20–30). Another survivor’s perspective illustrated the power of these agencies to commit extrajudicial crimes on innocent civilians: “*All the security branches have a very bad reputation—there are a lot of people who die [at intelligence branches]. It’s on a daily basis, it’s in a way that is arbitrary, or random—without any cause or based on the smallest reasons—maybe he was just arguing with his neighbor, and his neighbor reported on him. They take him, someone who doesn’t know what is going on, they hit him on his head, and they kill him. Just like that. It’s that simple*” (age range 40–50).

#### Captivity

The conditions, punishments, pain inflicted, abuse, and duration of captivity endured by survivors were heterogeneous, but certain patterns were identified. All survivors were held in miserable, cramped facilities where they suffered similar patterns and methods of torture.

**Conditions in captivity** The environment inside detention facilities was by itself a mode of torture, and it provided little comfort to captives between episodes of aggressive interrogations. These brutal environmental conditions threatened captives’ survival as they manifested in extreme starvation, overcrowding, sleep deprivation, denial of medical care, dreadful sanitation and hygiene conditions, chronic darkness, lack of ventilation, denial of clothing, exposure to sounds of others being tortured, and extreme weather and temperature conditions. Survivors repeatedly described sharing cells with dozens of other captives, which forced them to stand continuously. Cellmates organized themselves by taking turns to sleep in brief shifts. One survivor explained how such conditions restricted even their basic needs: “*We would be folded over ourselves—I’m telling you there were 45 [people] in a 30 square-meter room… Out of this includes the bathroom in the same room, and out of that space was a spot where people took off their shoes, and out of that space was where they kept some of the food—because sometimes they wouldn’t bring food to people—they make a clean place like that to put some of the food. And they would leave a space for one or two people to sleep in turns. Like if two people would sleep now, then after them, two others would go to sleep for an hour or half an hour or something. It’s just [enough time] so a human could continue living*” (age range 20–30). Another survivor further elaborated the consequences of the extremely overcrowded facilities: “*The dungeon is like an underground grave*… *We were 115 people in a cell room of 6 m length and 4 m width. The room had a small restroom and a sink. The temperature of the room was intolerable [hot]. We stayed there with minimal clothes, and every 6 h, we would squeeze the water from our clothes because of the amount of sweat and heat, just from the breath of people inside. The ventilation, they would open the window of the cell’s door once a day, just to have some air entering the cell. There was no other ventilation option… There was no option that everyone would sleep at the same time… We would be sleeping on each other, on top of each other… And your priority in your life becomes to keep the tile that you have conquered to sleep. My number one priority was to sleep*” (age range 20–30).

Food and sanitation were also extremely limited in all facilities. One participant described pests in her cell: “*I stayed for three days. Three days detained in a room. May God prevent you from seeing such an image. [It was] filled with lice*” (age range 40–50). After a period of detention in harsh conditions, captives experienced physical transformations. Another participant described the appearance of other prisoners: “*I could smell the flesh and blood. It was like their flesh was rotten. The situation was that 4 people had been stuffed inside [the cell]. Compressed, hot, there was no air. Barely any food. They would fight over the food they brought them. Animals don’t live in such conditions. When they released them, they were flea infested and filthy… They would be like skeletons because they barely gave them any food. You would see the person and his head was this size [big] and his body was this small. So small. Only skin on his bones, it was clear that they were inside [the solitary confinement] for a long period. Who knows [how long]. Maybe [for] four to five months and then he became a skeleton. Completely a skeleton*” (age range 30–40).

Captives in shared cells also had to endure regular cursing, humiliation, and collective beatings. A participant shared: “*That kind of thing [random beating] was not always clearly directed at one person. You wouldn’t know if they are directing it at you because, when there are 50 or 100 other persons crammed in with you, [bitter laughter] then there is no way to know which insult is intended for which person*” (age range 30–40). When not being directly tortured, detainees in their cells could often hear the sounds of others being tortured. One of the survivors expressed the impact of such terrorizing atmosphere: “*What was harder than everything, was the sounds of the people being tortured. We don’t see anything when we are in the dungeons. We can only hear the sounds*” (age range 40–50).

**Torture methods** Torture methods varied among facilities, guards, and captives. However, a common ground of all practices was their inhumane treatment and cruel purpose of inflicting pain, implementing punishment, and breaking the souls of inmates. Participants described the following diverse torture methods the regime apparatus practiced against its own citizens.

*Physical torture* All survivors reported enduring physical torture, which persisted throughout their captivity. As a standard operating procedure, guards and interrogators administered regular beatings to every prisoner: “*The tying of hands and feet, and the blindfolding… The general hitting, with sticks, batons, electrical cables, and such, were [used] for everyone*” (age range 40–50). Interrogators also employed more severe torture with individuals. One of the survivors described torture with electrocution: “*The harawan [baton] is a stick [similar to what] the police hold, it is rounded and has three buttons, with electricity. Imagine when my clothes would be wet, after they would wake me up [after fainting], they would hit me with electricity, then I would pass out [faint] again. Sometimes this torture would last for an hour, sometimes for two hours, and sometimes for more time*”. This same survivor also endured bisat al rih or the flying carpet: “*I was finished, I was done, they picked me up, I had no strength to move, and they pressed the wooden board tightly on me like this [a book], imagine to yourself that you were placed on your stomach and they brought the wooden board and pressed in on you like this [demonstrating], of course they didn’t reach all the way to the end, but I felt my back crack*” (age range 40–50). Some interrogation sessions involved mixed methods of abuse. Another survivor described enduring several punishments after angering her interrogators: “*They put my head in a small tub and tried drowning me. I would lose consciousness and would wake up to find myself lying on the floor, all wet. Then the interrogation would continue with electric shocks while being tied to the “doulab” [tire], and whipping and more electric shocks while I was on the doulab… [They were also] touching my body, my genitals and threatening to rape me, using their fingers and hands. I once spit at the officer who was interrogating me after sexually assaulting me, and he went crazy with the beatings and whippings and electric shocks. In that interrogation, he had ordered the other soldier who was with him in the room to pull out my nails*” (age range 20–30).

Many captives suffered from body mutilation like the second survivor in the above paragraph who experienced nail pulling. The first survivor above, on the other hand, witnessed the horrendous genital mutilation of three inmates who were hung naked on an iron frame, which resulted in their last breath: “*Each had a pool of blood dripping underneath them… They were mutilated, their penises were cut off*” (age range 40–50).

*Psychological torture: Threats and involvement of family and friends* Verbal assaults, witnessing the blood-stained facilities where other inmates were tortured before them, going through repetitive aggressive interrogations, and living in continuous uncertainty about when, where, and how they are going to be tortured were among a few methods causing psychological torture. However, among the most anxiety-provoking methods were threats to family and friends during interrogations that were commonly recounted by survivors. These threats served as psychological torture to encourage false confessions or to inflict further mental suffering. In addition to threats, several survivors indeed witnessed the capture, torture, or death of their loved ones. One of the participants described the unreserved use of such threats: “*During the interrogation, they would also threaten me, to bring my sisters and rape them, immediately, immediately they have this tactic*” (age range 20–30). Another participant, who was released after his parents had bribed officials on his behalf, was beaten while his mother listened on the phone: “*The intelligence took me in, and the head of the investigation branch was the first one to see me… He grabbed me and started beating me. My mother was calling me at the same time… He picked up the phone call [without my knowledge at the time] and put the phone on the table and started beating me and let my mother listen*” (age range 20–30).

These threats, even when not fulfilled, had significant impacts on the psyche of captives. One survivor, a mother, became consumed by the threats against her daughters: “*This was the strongest hit. I told them ‘My daughters are still children, and they would not understand what you are saying, and it's up to you if you want to bring my daughters or not’… So, to threaten me with my girls was what broke my back the most [knockout]. All night I would listen to the torture rooms, to check if there were any children's voices, a girl’s voice, or if there's anything like this*” (age range 40–50). For others, threats were brutally carried out. Another survivor was detained together with his brother and best friend, all students who were demonstrating for freedom. During investigation, the guards forced him to witness the torture of his brother in which they broke his brother’s nose: “*My brother was shouting, and blood was dripping from him. I was like this and my brother’s face next to me was like this, and his face was dripping with blood. Beating, beating, beating, whipping, whipping, and whipping… They said, ‘oh now we are going to make you rest even more,’ and they brought a cigarette and burned his hand*” (age range 20–30). During one of the participant’s interrogations and torture sessions, she witnessed the barbarian torture and brutal death of her best friend after the interrogators failed in coercing a confession from either one of them: “*When I refused to confess, they brought into the room my best friend [a young man 18 years of age] and tortured him in front of my eyes… They continued beating him, hanging him from the ceiling [al-shabeh], and whipping him… They nailed him to the wall in chains and electrified him over and over, while yelling at him to speak and he would say ‘I don’t know’. They told me that if I confessed about what he has done or what I have done, they would stop torturing him. He would cry out loud to me, ‘don’t tell them anything, don’t even think to say a word to make it easier on me… They will do it their way no matter what you did or said’… When they didn’t get any response from me other than ‘I don’t know’, they went to him again and started cutting into his skin with a blade, cutting and cutting and then putting the electric cables into the cuts and electrified him. At the end, they just hung him from the ceiling and cut his fingers with an electric saw in front of my eyes and then they cut his genitals with the same saw and let him bleed to death [she started crying after she finished with this description]*” (age range 20–30).

*Depersonalization, derealization, and solitary confinement* Several survivors reported experiencing episodes of depersonalization and derealization while undergoing intensive torture, as well as during prolonged periods of solitary confinement. Phenotypes of depersonalization and derealization included feeling entirely disconnected from oneself, the inability to perceive elapsing time, the inability to recall one’s identity, difficulty ascertaining what is real, and a dissociative state in which one witnesses their body being tortured from an external perspective. Lengthy periods of solitary confinement were especially prone to triggering depersonalization, which may be interrupted only by episodes of physical torture.

One of the survivors explained how solitary confinement was used during her captivity: “*They would drag me because I wasn’t able to walk on my feet… They would put me alone [in a room]. They would leave me for one day, sometimes for 2 days, and then they would bring me back [out] again and say… ‘You’re going to talk, right?’ I would say ‘I swear [wallah] there is nothing for me to confess’, and they would do the same torture all over again. As much as I swore, I would tell them ‘For God's sake, please, I am pregnant, have mercy on me a bit,’ they would say ‘Who is your God [Allah]? Our God is Bashar. Who is your [plural] God?’ One time I told them, ‘Our God is the one who created both of us, this is our God.’ I stayed in solitary confinement for 12 days, where I didn't see the light, and would not drink water or anything. Deprived from anything at all. Just torture. They would torture me without [asking] any questions and then send me back again to the solitary confinement*” (age range 40–50). The depersonalization experienced during solitary confinement was described by another survivor: “*They took my cell phone and my cloths, and left me with my underwear… I didn’t know it was two months that had passed, because when you are under the ground and you don’t see the daylight, it is hard to distinguish when a day started and when it ended. You lose all sense of time whatsoever. I knew two months had passed only when I reached the new facility and I'd been told in there about the date*” (age range 20–30). Loss of perception of time was especially damaging for one of the participants, who said: “*The environment was dark, but a person, in their mind, gets a black wall in their mind [blackout]. A person can’t imagine what is happening anymore. Now, I—the problem was that I no longer had any perception of time—it all changed for me—two days, three, or four, I didn’t know what sleep was anymore. I forgot.*” This participant had also experienced difficulty in ascertaining what was real and unreal during investigations. In one of the events, he narrated: *“I started doubting myself… I started suspecting them [friends] and everything… Is it really possible they betrayed me that easily? Why me, specifically?*” On another occasion he said “*I thought I was lying to myself, is it possible that I have been here for 9 nine days or was this a nightmare?… Like all that happened was within my imagination and it wasn’t a reality?*” (age range 40–50).

*Positional torture—Stress position abuse* Diverse variations of positional torture were also reported, in which captives were forced to be in fixed positions for extended periods of time. One survivor was forced to kneel for prolonged periods: “*I would tell him [the guard], ‘For the love of God, slaughter me, beat me, but don’t make me sit like this’. The knee would get sculpted to the inside [the bone of the knee gets inside the body]. Like when you put a stone like this and it gets inside the place [the knee bone]*” (age range 30–40). Another survivor was made to stand for ten days and kept awake with hypothermic torture: “*He [the guard] tied me to an iron pole, with my hands handcuffed behind me and my eyes blindfolded. I was in my underwear and in front of me there was a big mobile air conditioner… It was to this height [waist], and I was standing in front of this air conditioner for ten days, and it was forbidden to sleep. How would they know that I was sleeping [under blindfolds]? Whenever my neck would drop down, they would pour water on me. I would shiver to an extent that my thigh bone would cramp with uncontrollable intensity, and I would have compulsive cramps in my kidney because of the cold. I broke two molar teeth from shivering. As soon as my body would get adjusted to the temperature, he would pour water on me to make me shiver again… You would go to the restroom once a day and you cannot ask to go to the restroom whenever you need it… They have no problem if you pee on yourself*” (age range 20–30).

*Al-Shabeh—Hanging* ‘Al-Shabeh’ (the phantom or hanging) was described as an especially notorious method, which combined brutal beatings and positional torture. A participant explained this almost ritualistic method: “*The phantom [al-shabeh], which was when they would tie a person by their hands… He would remain hanging by his hands, and they would hit him after they torture him*” (age range 40–50). Another participant survived this method and said: “*Once they hung me up [al-shabeh] in that prison in Damascus… They hang a person from their hands… Their feet don’t touch the ground and they keep them like that for a few days… People stay [hung] two months or three months. Some people die while standing [in this position]… They hit me with the whip and that pipe… Or, for example, if a person faints [while hanging] or needs to use the restroom [while hanging] they beat them… They brought handcuffs and they hung the person from them on a metal bar… The person hangs and the tips of their toes barely touch the ground. I entered a room that was about this size and everyone was hung up [in different locations of the room] and they hung me in one spot. Of course, we were all blindfolded. But I could feel that there were other people in the room*” (age range 30–40). The first participant above was hung by his leg for several days in an upside-down position: “*While I was hanging by my leg, I used to lose consciousness. Once, twice, three times, I don’t know. I don’t remember [how many times]*” (age range 40–50).

*Rape by interrogators* Rape, or the threat of rape, was frequently and especially used against female captives. Rape in the Arab society has disgraceful implications not only on the woman’s reputation and marital prospects, but also on the honor of her family and thus, “sexual abuse remains a mechanism to oppress, suppress, and deny women of their humanity” ([35], p.1290). One participant described the widespread use of rape threats: “*The female prisoners, when they get imprisoned, the first topic they get threatened with is their virginity… ‘Now we will rape you, now we will do this to you, and now will do that to you*’ [imitating the threats]” (age range 20–30). Another participant suffered immensely in trying to protect her daughter from being raped during their joint captivity: “*When they were trying to rape her or [tried] bringing men in the room to rape her. I kneeled at the feet of the officer and told him, ‘I will kiss your feet, take what you want from me, but not my daughter, don’t hurt my daughter, I swear she has nothing to do with this’ [crying]”* (age range 40–50). One survivor witnessed the repeated group rape of a detained gynecologist in the adjacent cell: “*At first, we used to hear her screams while they raped her and humiliated her and laughed at her and threatened her, all four or five of them together. We would hear their laughter and joy while [they were] ‘partying’. But afterward [with time], we would no longer hear her while they continued raping her. She was totally in silence. Muted, numb, as if she didn’t feel anything anymore and as if she wasn’t there anymore… She would be forgotten in her cell [after the rape], thrown at the floor bleeding to death the whole day, until they came to take her the next day and group rape her again*” (age range 20–30).

*Prisoners forced to rape other prisoners* In another form of sexual violence, guards orchestrated and forced rapes between prisoners as a demonstration of power. One participant described how guards forced prisoners to watch as other prisoners were forced to rape each other: “*In front of everyone… Like we were watching… ‘If you do this, then this is what will happen to you.’ They brought him (another prisoner) from another cell–I don’t know from where–and they told them, ‘Now, you need to have sex with each other.’ And they had sex, and they didn’t dare say anything. And [the guards would say], ‘don't cry, you have to enjoy it. You have to act like you’re enjoying it’ while beating both of them. This re-occurred over the course of the four days at the same place… [We] were naked*” (age range 40–50).

*Killing of prisoners* In telling their own stories, survivors also related seeing or hearing guards execute or kill other detainees. As part of her own interrogation, one of the participants was made to watch the torture and eventual killing of her friend: “*After they killed my best friend in front of my eyes, I fainted. When I woke up, all my body was covered with blood, the room was full of blood, it was like a pool of blood on the floor and he [my friend] was still hanging, without a breath in his soul, still dripping so much blood from his body*” (age range 20–30). Another participant would see the bodies of other prisoners during his captivity: “*When we used to walk in the hallways, we used to see bodies rapped in carpets, people who died *via *torture*” (age range 20–30).

*Health related torture* The regime not only practiced torture methods within detention facilities and prisons, but also in military hospitals. Survivors testified to being denied basic medical care, and to being subjected to abusive medical experiments and procedures in the regime’s military hospitals and detention facilities. Physicians and nurses who worked in military hospitals also witnessed and described horrifying practices during their work.

One participant described how the guards actively prevented him from taking his daily medication: “*They brought me my medicine. The same medicine I took in Daraa, they brought [to Damascus]. The soldiers in Damascus were dirty and did not give it to me. I told him I want my medicine and he didn’t agree to give it to me. And I knew it was with him, he showed it to me. He said, ‘Are you addicted to the medicine [getting high from the medicine]? You can’t have it’ [and hid the medication]*” (age range 30–40). Another participant was deliberately given psychoactive drugs, which she was told would help her with the pain she endured: “*These 3 pills, I was required to take daily. When I had them, I would forget, I would forget they had beaten me, I would forget they had hit me, I would forget anything that had happened to me… It was obligatory to take them… They would hand them to me daily, from their hands.*” However, when later on she was examined by Jordanian doctors, the truth about the pills and the medical experiment she went through was revealed “*[doctors said] ‘These cause a condition of a memory loss, not a normal one, to the extent of hallucinations’, so that I get out of their prison done*” (age range 40–50). One of the survivors, who previously served as a military physician, attested to the regime’s maltreatment of captives and soldiers: “*Someone [soldier] would have a stomach ache, and they admitted him [to the hospital] on the basis of appendicitis. They ripped his stomach, and they took his kidney, and they took him back to the base… So incidentally, once upon a time, someone came in with appendicitis symptoms. I was in an ambulance shift… As an emergency physician… I told them to… run the tests for him because it seemed from my examination in the ambulance that this was appendicitis, so he [soldier] told me: ‘No doctor, I [already] had an operation for appendicitis in the past.’ He was [still] hurting, and these were 100% appendicitis symptoms… They [eventually] got him in for an operation, and it was appendicitis. They [the military physicians] had indeed removed his kidney and not the appendix*” (age range 40–50).

#### Post-captivity and the aftermath of torture

Survivors were ultimately released from captivity after varying lengths of detention. New challenges arose as they attempted to return to civil and domestic life while coping with the extreme trauma they were exposed to during captivity. Survivors provided some common insights about their escape journeys and individual reintegration experiences. They also reflected on their attempts to establish new lives in Jordan.

**Release from captivity** Several survivors were released with the fortunate help of relatives who afforded the necessary bribes required by prison officials. When her family secured a bribe, one of the participants received better treatment, was spared additional torture, and was soon released: “*They [the police] were very happy, because the police took [money]. That’s how they treated me. They all treated me like this because of the money, all because of [bribe]*” (age range 40–50). In addition to bribes, connections were also needed to secure another survivor’s release due to the severity of the claims fabricated against him: “*My dad came and he brought people he knew [his connections] to the head of the [intelligence] branch. He fixed everything, and he [the father] was the one who got me released… They would not have released me [without bribery]… He [family’s friend] had arranged everything for me with my dad because he knew important officers*” (age range 30–40). Another participant was also released after his family paid prison officials a bribe: “*I left prison from the front door of the branch. I didn’t [need to] go to the court [first]. My father paid a decent amount of money and gold to release me*” (age range 20–30).

**Unreleased captives after enforced disappearance** Many detainees, whose families had limited resources leaving them unable to participate in the incentivized corrupted system, remained incarcerated, while the survivors of this study were released. These families were yearning for any information regarding their loved ones. A participant described the complex situation “*There are still thousands inside… Some people [relatives of detainees] receive news about them being executed, some people say they stayed in jail and are still alive. No one really knows anything. Some people keep hoping [that their loved ones would leave]. Some people are already killed and buried, and all done, but some people continue hoping that [their loved ones] are still alive… God knows where they threw them and where they buried them*” (age range 30–40). In the setting of the regime’s restriction on communication and reporting, captives tried to communicate with the families of co-captives met in detainment and helped each other with delivering information, as one survivor explained “*We used to give each other our numbers, and we used to sew it inside the cloths… On the stitch of the inside of the pants so that they [guards] won’t see it, we would write the [phone] numbers in sewing, so that maybe one of the girls was released from detention, and she could inform our families where we were being held, because no one knew where we were*” (age range 40–50). Such collaboration between detainees represents the complexity in delivering information to the outside world and the refusal of the regime to acknowledge the fate of people it had arrested, detained, or abducted, in violation of fundamental human rights.

**Feelings upon release** Survivors reported intense euphoric emotions, mixed with fear, disbelief, excitement, anticipation, and new appreciation for their freedom upon release from captivity. Some survivors narrated that they were in shock upon release, whereas others felt ashamed from the way they looked and smelled when they first met their families. One of the participants compared his release to a resurrection: “*Oh I was so happy. I forgot all the beating and everything that I went through once I saw life and my family and my kids and such… How difficult it is for a person to return and see life [on the outside]! This period of 5 months [length of captivity] was like dying and then returning to life… Then coming out and seeing people walking, eating, and smelling good scents [disbelief]*” (age range 30–40). Another survivor experienced heightened appreciation for the simple pleasures in daily life: “*It [falafel] smells so good. I used to miss it so much. You would miss things you have never had thought you would miss and appreciate. The food, clean water, showering, sleeping, seeing friends, enjoying other people’s company, just walking in the street, going to buy some groceries. The most common things. Normal life. Because down there [dungeons], there is nothing normal that any human mind can comprehend, ever*” (age range 20–30).

**Escaping Syria** After their time in Syrian dungeons, survivors had little chance of regaining any pre-captivity normalcy. They were labelled as ex-prisoners in the Syrian system, so they faced increased suspicion from authorities, and thus they were at increased risk of recapture and re-torture. This was a chief motivator for survivors to seek refuge in Jordan immediately after their release. One participant left for Jordan only hours after his release, “*My neighbor arrived at our house and told me, ‘Don’t stay here. There was a shooting at the checkpoint, and they heard you were released. They think you shot at them. They will take you in the morning if you stay here.’ In the morning, at 6AM, as soon as the light came out, my dad brought me a car and took me to Naeemeh, which is close to the border…So I could enter legally*” (age range 30–40). Another participant also swiftly escaped Syria with her daughter after they were released: “*They let us out of the detention facility at 3AM, my daughter and I... We took a shower, ate food, and I stayed asleep until 6AM or 7AM. Then I called my parents to let them know that I was out… Then the next day at 10AM, I called a driver who is one of my relatives… I told him, ‘I want to go out,’ and he got me out and I left [Syria]*” (age range 40–50).

**Life in Jordan** Survivors spoke of new adversity and a difficulty assimilating in Jordan, both emotionally and financially. One survivor even attempted to return to Syria due to her new financial woes, however, the hovering threat to herself and her family of being targeted and recaptured forced her to consider migrating outside of Jordan: “*I tried to enter inside [Syria], to go back. And, until this moment, I'm very determined. Because, regardless of anything, a person in their own homeland is something different. Even if it is under shelling it is still better [than being displaced]… It's a very hard situation being here [Jordan], in all honesty... I'm afraid that we are being chased and followed until now. This is what scares me. Therefore, I'm thinking about leaving Jordan, meaning to migrate outside of Jordan*” (age range 40–50). Another survivor endured the hardships of displacement in Jordan because he was too fearful of being detained again in Syria: “*We became refugees here… We lost a lot of money… But I could tolerate it here [more than his wife] because she didn’t see what I saw [experienced] in prison. If I could, I would go back right away to my home [in Syria], but it’s impossible. I can’t return and [risk] them arresting me again. It’s impossible*” (age range 30–40). A participant attested to his economic hardship due to the limits of work permits for Syrians in Jordan: “*I rely on friends' assistance to pay the rent and survival needs*” (age range 40–50). Initially, another participant experienced a phase of total rejection for his new surroundings: “*This was my first-time leaving Syria in my life. I couldn’t imagine leaving Syria in the first place, I stayed the first six months in Jordan… [Something] in my subconsciousness was refusing to learn the streets [names]. When the snow came down… Even when it melted, I didn't leave [the house because] I won't leave. I won’t touch snow except in my country [Syria]*” (age range 20–30).

**Political activism** Multiple survivors became motivated to participate in activism and volunteerism post-displacement in Jordan. One survivor joined a relief effort serving other refugees: “*I worked for three months in relief. One of them was the last month of Ramadan, in 2013. In the month of Ramadan, I distributed 3000 food baskets through the activism [effort] of our group*” (age range 20–30). Another survivor also became involved in serving Syrian children in Daraa while being in Jordan: “*I am involved in educational activities for the Syrian children with local humanitarian organizations (Daraa)… We have proposals and everything ready, but we need donors to help in implementing them… We have a newsletter where we post our activities, food distribution, providing school supplies, cleaning the garbage from the streets, educational activities in classes for the children, music, and art classes, *etc*.*” (age range 20–30). The spirit of resilience and self-reliance, voiced by many survivors were also complemented by reflections on the social and political situation as attested by one of the participants: “*[Calm voice] It is very necessary to deliver a message to our Arab world so it changes its way of thinking… We need to be more open, become more open to people, become more open with our own internal world, to listen a little bit more to ourselves, and listen to the children within us*” (age range 40–50). Others however, chose to return to Syria and joined armed groups who were fighting the regime in the hope to free Syria “*Our orientation is to make Syria a civil country in the future and a democratic country, different than what exists now, which is based on all freedoms, and is open to all kinds of freedoms*” (age range 40–50). Others chose to be engaged in the rescue of fighters who were injured and needed transportation from the borders to hospitals in Jordan. One of the survivors witnessed many of his friends’ deaths, “*One time I went back to bury my friend, I went and washed [ghusl, before burial] my friend and came back… My friend’s brain… his brain, I put it in a bag. He got hit by a missile shrapnel entering from here [gestures direction] and it [shrapnel] got out of his head. His brain was splattered everywhere. When we washed him and got him ready, I carried his brain and put it in a bag. We put the body in a car, and we went to give him off [to his family]… At the beginning you would get emotional and then you cry and then after that it’s fine*” (age range 20–30).

## Discussion

This study bears witness to the many survivors' abhorrent testimonies of the extreme atrocities and cruelty of an organized and systematic apparatus of torture and surveillance in Syria. The presented subjective severe experiences of survivors testify that the Syrian regime operates an initial detection and arrest system, as well as an intelligence system with multiple detention facilities partitioned among its four major government agencies—military, political, air force, and state security. Each agency has its own branches wherein it specializes in the investigation of certain allegations. Many individual branches carry a reputation associated with their predilection for severe torture or death. This prison network is easily accessible to local officials throughout the country who can capture and detain civilians without evidence or any kind of judicial oversight. Thus, ordinary Syrians are cautiously aware of the low threshold for imprisonment [36, 37]. Survivors described being abducted arbitrarily in their homes, on their way to work, or while demonstrating in the streets, without any provocation. Many experienced enforced disappearance, during which they were captured against their will, and their families had no idea where they were held and if they were still alive; authorities would not reassure families of their loved ones’ fate, or even acknowledge their captivity at all.

Once detained, captives were immediately bound and transported to facilities. The authorities facilitating their capture often did not provide official warrants or charges upon arrest, and captives had only learnt of allegations made against them during interrogations. Beatings began at the onset of capture and climaxed with a “welcoming party” or “reception party” upon arrival to a detention facility [12]. Throughout detention, prisoners would endure frequent and daily torture both in interrogations and in their cells. Routine mass beatings were frequently employed by the guards. Captives accused of multiple allegations found themselves transferred to a series of other branches in the network, where they endured additional interrogations. Captives may have been fortunate to be released after days, months, or years, especially if their friends and family were able to secure bribes or connections [38]. At other times, captives reported being randomly released, without any specific allegation or reason to be released, except to seemingly create a “living example” as a form of public intimidation [37]. However, participants also witnessed others who would find their death during captivity without leaving a trace, or even an official record that can be delivered to their families. Many others who shared cells with survivors remain detained and continue to face torture in the regime’s numerous facilities with no anticipated time or hope for release.

Interrogators across the facilities employed multiple methods of torture in punishing and inflicting physical and psychological pain onto captives. Captives were almost always bound, blindfolded, and naked during detention. Torture methods encompassed beatings, flogging, dragging, electrocution, and the witnessing of killing, sexual assault, or torture of others. These may be combined with positional abuse methods, which have been defined in various terms, such as *bisat al rih* (flying carpet), *dulab* (tire), *falqa* (feet whipping), *al-kursi al-almani* (the German chair), *al salb* (crucifixion), *al-shabeh* (hanging), burning, hot/cold water pouring, drowning, and suffocation. Digits, genitals, and other sensitive body parts were frequently mutilated or severed. Sexual violence was also common and included threats to the prisoner or their families, rape by guards, and prisoners being forced to rape each other. Mass killings of captives were very frequent, as well as health related torture, which included denial of basic medical care, and abusive medical experiments and procedures in the regime’s military hospitals and detention facilities.

The Syrian Network for Human Rights (SNHR) [14] has documented 72 torture methods that the Syrian regime practices in detention centers and military hospitals, most of which were experienced and reported by survivors in this study. In addition, mental and emotional methods of torture included the involvement of family and friends as targets of threats and violence in trying to intimidate captives and coerce false confessions. More routinely, prisoners were deprived of sufficient cell space, sleep, food, warmth, and sunlight. Medicine and medical care were withheld, malnutrition and starvation were prevalent, and disease was allowed to spread between prisoners. The combination of torture and poor environmental conditions created a sense of timelessness, helplessness, and misery among captives. Through this brutal treatment, interrogators would hope to break the spirit of their captives, silence their voices, and thus establish a regimen of control, oppression, dominance, and blind obedience [12, 37].

## Recommendations

The use of torture and extrajudicial captivity in Syria have long been acknowledged among international bodies such as the UN agencies [20]. Given the current complex geopolitical situation in Syria, there are no clear avenues for direct interventions to-date. At the minimum, there is a global obligation to bring justice for the victims of torture by pursuing legal prosecution of all responsible parties. The stories of Syrian survivors are largely unheard. To that end, more studies are needed to report on the state of torture in the Syrian detention system. Furthermore, there is an immense psychological burden among Syrian refugees related to torture. Many Syrian refugees are either torture survivors themselves or have spouses, children, relatives, or friends who have been detained or tortured [39–42]. For the survivors interviewed in this study, torture changed their lives immediately and produced ramifications extending into their refugeehood. As several survivors noted, there were consequences within the stages of captivity and post-captivity, which disrupted relationships with employers, loved ones [43], and their surroundings, but mainly with themselves. While all survivors were fortunate to escape captivity and torture, they were left to internalize personal stigma while also suffering from social stigma, leading to further isolation and trauma. It is critical to share and disseminate their stories so we may improve our understanding of their experiences and better address their needs.

One challenge of producing and disseminating this type of study has been the fear of retaliation from the Syrian security apparatus through its international network of informants. Both the interviewed survivors and this study’s authors experienced safety concerns during their involvement in producing this work. This concern has likely contributed to the dearth of similar studies [33]. It is the belief of the authors that the need to study this issue from the perspective of torture survivors outweighs the risks taken. Our hope is to increase the understanding of the complex and accumulative traumas, and human rights violations experienced by many Syrians, and to deliver their silenced voices among the international community to support efforts in achieving freedom, democracy, justice, and equality for the thousands still imprisoned, as well as their tormented families. It is also hoped that participation in this study would empower survivors in advocating for themselves and presenting their subjective stories, which may advance their healing and recovery process.

## Limitations

A few limitations were observed in this study. The analysis did not explore how the researcher’s presence impacted participants’ narratives. Interviews may have been influenced by gender dynamics between the researcher, who is a woman, and the majority of the torture survivors interviewed who were men. These gender dynamics may have increased or decreased participants’ comfort and ability to disclose certain experiences, such as male sexual assaults, or other humiliating or degrading acts that are considered taboo topics, especially in the Arab society [35]. Furthermore, the analysis did not consider the group interpretive processes and the richness of their perspectives, given that most of the research team is Syrian [31]. Thus, in addition to content and form, future research should also address the co-construction of narratives in torture-related studies. Finally, the study has a sample selection bias. It is limited by its small sample size (especially women participants) and heterogeneity (i.e., Syrians, refugees, torture survivors, etc.), as well as the specific time (in 2014) and space (Jordan) in which the interviews took place. Study participant recruitment occurred via a snowball sampling process during a brief period of time due to the limited duration of the first author’s trip to Jordan. Nonetheless, it is very rare to have survivors of torture agree to participate in research studies due to the social stigma from which they suffer, and the hovering threat to their lives if confidentiality might have been breached or compromised. It could be that survivors who endured torture in recent years or who had escaped to different neighboring countries other than Jordan would have had different personal narrations related to their subjective experiences with the Syrian regime’s detention facilities. Moreover, the researcher was unable to conduct more than two interviews per day due to the diverse geographic locations in Jordan where participants resided, and the secondary traumatization involved given the sensitive topic of these interviews [32].

Despite these limitations, this study fills an important knowledge gap on Syrian torture survivors and their personal and collective experiences before and during captivity, and the impact of their suffering on post-displacement life and activism. Their painful experiences and abuses are crucial for the international community to acknowledge amidst the ongoing complex war in Syria and its associated brutal human rights violations. It may impose some pressure to internationally prosecute the regime for war crimes, release information on the fate of the disappeared, reveal the location of the buried, and to allow families to visit those still alive or at least reunite with their remains. Such prosecutions or reforms are hoped to bring justice and assist survivors with the restoration of a new life.

## Conclusions

The shared experiences of torture survivors reflect the systematic implementation of transgressive practices of torture across Syrian detention facilities. The idea that a civilian can be readily imprisoned and punished extrajudicially permeates Syrian life. Imprisonment and torture by authorities are commonplace themes displayed in Syrian television, cartoons, and jokes, as they have been serving as symbols of social control and power through two generations of rule under the al-Assad family [36]. This apparatus serves two purposes, which the Syrian regime views as essential to its survival as a dictatorship. First, the ever-present fear of detention suppresses dissent and preserves the ruling elite. Well-connected groups possess considerable power to readily report unfavorable individuals, and they can wield this influence over the masses. In other words, the Syrian regime uses torture as a counterinsurgency method on its home soil [37], wherein fear and involuntary compliance are essential to the regime’s existence as a state built on corruption and subjugation [36]. Survivors in this study articulated this point vividly and repeatedly utilizing the phrase “a regime that has ears and arms everywhere.” Second, the detention system is an industry that encourages the large-scale fabrication of allegations and the production of confessions of serious crimes. Branches are motivated to produce more confessions so they may demonstrate value to their superiors and extort additional funding to support the regime and finance the infrastructure of detainment [38]. This propels the system’s brutality further and contributes to the state’s violence compliance and complicity of authorities at every level of the detention process. This second point was also present in survivors’ testimonies when they tried to understand the reason for enduring such suffering in their own country by their own people.

Survivors in this study narrated harrowing accounts of torture at several facilities during detention. They all described the suffocation from overcrowding and the degrading conditions, mass beatings, and a variety of severe torture methods. The intelligence system seems to have reacted to protests and dissent by ramping up collective punishment on the Syrian population. Survivors witnessed and heard about the execution of other prisoners, and other reports have shed light on specialized facilities where thousands of Syrians were likely exterminated [18, 21]. The institutionalized system described may be disturbing to outsiders, but it is notorious among Syrian civilians and well-known in the Arab region. Unfortunately, this topic has received relatively little public and academic attention, as well as minimal funding in resources for further investigations.

Despite the extreme and severe scale of imprisonment and torture in Syria, there have been only a few published works analyzing the regime’s systemic use of torture. In 2014, the Caesar Report, the largest report published so far, provided the most extensive photography collection of proof sourced from an anonymous former Syrian forensic investigator who worked with the Syrian military police and leaked thousands of photos of civilian detainees massacred and tortured within military execution prisons [17, 44]. While the Caesar report and other works from humanitarian organizations have provided concrete evidence of torture, and emotionally moved the international community, the personal voices and narratives of survivors have largely not yet been heard. This study appears to be one of the few academic qualitative analysis of interviews with individuals who experienced and survived torture in Syria. It is also unique in analyzing the common themes experienced before, during, and after captivity, as they were personally narrated by survivors, and in the form they contextualized and wished for the world to hear and acknowledge them. This analysis sheds light on the structure and function of the intelligence system and its widespread use of particularly cruel torture methods. Additionally, this study highlights survivors' humanity, resilience, and persistent challenges, which are indeed relevant to the international community.

## Data Availability

The datasets generated and/or analyzed during the current study are not publicly available due to their sensitivity as they may contain information that could compromise the participants’ privacy and impose risk to their lives; but are available from the corresponding author on reasonable request.
